# Intravenous administration of *Streptococcus mutans* induces IgA nephropathy-like lesions

**DOI:** 10.1007/s10157-020-01961-1

**Published:** 2020-09-09

**Authors:** Shuhei Naka, Kaoruko Wato, Taro Misaki, Seigo Ito, Yasuyuki Nagasawa, Ryota Nomura, Michiyo Matsumoto-Nakano, Kazuhiko Nakano

**Affiliations:** 1 Department of Pediatric Dentistry, Okayama University Graduate School of Medicine, Dentistry and Pharmaceutical Sciences, Okayama, Okayama Japan; 2 Division of Oral Infection and Disease Control, Department of Pediatric Dentistry, Osaka University Graduate School of Dentistry, Suita, Osaka Japan; 3 Division of Nephrology, Seirei Hamamatsu General Hospital, Hamamatsu, Shizuoka Japan; 4 Department of Nursing, Faculty of Nursing, Seirei Christopher University, Hamamatsu, Shizuoka Japan; 5 Department of Nephrology and Endocrinology, National Defense Medical College, Tokorozawa, Saitama Japan; 6 Division of Kidney and Dialysis, Department of Internal Medicine, Hyogo College of Medicine, Nishinomiya, Hyogo Japan

**Keywords:** IgA nephropathy, Intravenous administration, *Streptococcus mutans*, Dental caries, Rats, Glomerulonephritis

## Abstract

**Background:**

IgA nephropathy (IgAN) is one of the most frequently occurring types of chronic glomerulonephritis. Previous analyses have revealed that a major pathogen of dental caries, *Streptococcus mutans* [which expresses collagen-binding protein (Cnm) on its surface], is involved in the pathogenesis of IgAN.

**Methods:**

Cnm-positive *S. mutans* isolated from a patient with IgAN was intravenously administered to specific pathogen-free Sprague–Dawley rats to evaluate their kidney conditions.

**Results:**

The urinary protein level of the *S. mutans* group reached a plateau at 30 days, with increased numbers of mesangial cells and an increased mesangial matrix. The numbers of rats with IgA-positive and/or C3-positive glomeruli were significantly greater in the *S. mutans* group than in the control group at 45 days (*P* < 0.05). Electron microscopy analyses revealed electron-dense depositions in the mesangial area among rats in the *S. mutans* group. There were significantly more CD68-positive cells (macrophages) in the glomeruli of the *S. mutans* group than in the glomeruli of the control group during the late phase (*P* < 0.05), similar to the findings in patients with IgAN.

**Conclusion:**

Our results suggested that intravenous administration of Cnm-positive *S. mutans* caused transient induction of IgAN-like lesions in rats.

## Introduction

Immunoglobulin A nephropathy (IgAN) is the most common chronic form of primary glomerulonephritis [1, 2]. It has been reported that 30–40% of patients with IgAN exhibit progression to end-stage kidney disease within 20 years [1, 2]. However, there is no specific treatment for IgAN, because the underlying pathogenesis of the disease has not been fully elucidated [3]. The pathology of IgAN is known to be characterised by IgA deposition in glomerular mesangial cells. Some patients with IgAN (approximately 30%, primarily young individuals) have a clear clinical history of disease exacerbation after tonsillitis and upper respiratory or gastrointestinal infections [4, 5]. Several bacterial species have been proposed to be associated with the pathogenesis of IgAN [4, 6–10], indicating that infection is likely to play a major underlying role in some affected patients.

*Streptococcus mutans*, a Gram-positive facultative anaerobic bacterial species, is a major pathogen of human dental caries [11]. *S. mutans* strains expressing the collagen-binding protein (Cnm) on their cell surface exhibit a binding ability for the extracellular matrix; thus, Cnm may be a virulence factor in several diseases such as infective endocarditis [12], aggravated cerebral haemorrhaging [13–15], non-alcoholic steatohepatitis [16], and inflammatory bowel disease [11]. Furthermore, the results of our recent studies suggest that Cnm-positive *S. mutans* strains are associated with IgAN [17–19].

Several in vivo studies in the 1980s and 1990s hypothesised a correlation between *S. mutans* and nephritis [20, 21]; however, to the best of our knowledge, there have been no subsequent reports regarding this relationship. We recently found that the proportion of Cnm-positive *S. mutans* strains isolated from the oral cavity was significantly greater in patients with IgAN than in healthy controls; moreover, the presence of Cnm-positive *S. mutans* and the dental caries status was both associated with exacerbation of urinary protein levels in patients with IgAN [18]. However, the detailed mechanism associated with IgAN remains unknown, because no available experimental animal models have demonstrated that Cnm-positive *S. mutans* infection induces IgAN. Accordingly, this study was performed to investigate whether Cnm-positive *S. mutans* contribute to the onset of IgA-like renal lesions, and to establish a rat model of IgAN related to infection.

## Materials and methods

### Bacterial strain and culture medium

*S. mutans* strain SN74 (serotype *e*) was isolated from the oral cavity of a patient with severe IgAN about 5 years ago. The patient was 60 s. His serum creatinine level was 1.02 mg/dl, estimated glomerular filtration rate was 58 ml/min, and urinary protein grade was 1 + ; he had no urinary occult blood and no history of gross hematuria. The patient was prescribed a renin–angiotensin system inhibitor at that time. Approximately 15 years ago, the patient had undergone renal biopsy; the findings had supported a diagnosis of IgAN, with Oxford classification M0E1S1T1C1. At the time of diagnosis, his serum creatinine level was 1.12 mg/dl, estimated glomerular filtration rate was 54 ml/min, urinary protein grade was 3 + (2.93 g/g Cre), and urinary occult blood grade was 2 + . Steroid therapy was administered for 1 year about 15 years ago. The SN74 strain was confirmed to express Cnm on its cell surface. The strain was cultured on Mitis-Salivarius agar (Difco Laboratories, Detroit, MI, USA) plates containing bacitracin (0.2 U/ml; Sigma Chemical Co., St. Louis, MO, USA) or brain heart infusion (Difco) broth.

### Animal experiments

All rats were treated humanely, in accordance with National Institutes of Health and AERI-BBRI Animal Care and Use Committee guidelines. All procedures used in the present study were approved by the Animal Care and Use Committee of Okayama University. The effects of intravenous administration of *S. mutans* were analysed in a rat model, as described previously [22] with some modifications [11, 16, 23]. Briefly, specific pathogen-free Sprague–Dawley rats (male, 4 weeks old; Japan CLEA, Tokyo, Japan) were randomly divided into control and *S. mutans* groups. Rats were allowed free access to water and food throughout the experimental period. Rats were fed an MF diet (ORIENTAL YEAST CO., Ltd, Tokyo, Japan). Rats received intravenous injections (through the jugular vein) of *S. mutans* (1 × 10^8^ colony-forming units) suspended in 100 µl phosphate-buffered saline (PBS) or PBS alone (i.e., without added bacteria).

The rats were euthanised at 15, 30, 45, and 60 days after infection; their kidneys were then removed. Urinary levels of protein and creatinine were measured by Nagahama Lifescience (ORIENTAL YEAST CO., Ltd, Shiga, Japan). Serum levels of creatinine, albumin, and blood urea nitrogen were also measured by Nagahama Lifescience.

Tissue samples were fixed in 3.7% formaldehyde (diluted in PBS), embedded in paraffin, and cut into 3 µm-thick sections for histopathological analysis. Periodic acid-Schiff staining was performed to evaluate increases of mesangial cells and mesangial matrix in glomeruli. Mesangial proliferation scores were then calculated based on the proportion of glomeruli with mesangial cells and matrix proliferation among 50 glomeruli in Periodic acid–Schiff-stained sections. Additionally, alterations in IgA, C3, and CD34 expression patterns in tissue samples were detected using standard immunohistochemical techniques with IgA-, C3-, and CD34 (vascular endothelial cell marker)-specific antibodies. The primary antibodies used were Purified Mouse Anti-Rat IgA (BD Biosciences, Franklin Lakes, NJ, USA), anti-C3 (B-9) (sc-28294; Santa Cruz Biotechnology, Dallas, TX, USA), and anti-CD34 (EP373Y) (ab81289; Abcam, Cambridge, MA, USA) antibodies. Secondary antibodies were Donkey Anti-Mouse IgG H&L (Alexa Fluor 488) preadsorbed (ab150109; Abcam) and Donkey Anti-Rabbit IgG H&L (Alexa Fluor 647) (ab150075; Abcam). Fluorescence immunostaining was performed using these antibodies. Stained sections were observed using an all-in-one fluorescence microscope (BZ-X700; Keyence, Osaka, Japan).

The distributions of immunocompetent cells in the kidney were analysed by evaluating alterations of CD43 (neutrophil marker) and CD68 (macrophage marker) in tissue samples using standard immunohistochemical techniques with CD43- and CD68-specific antibodies, respectively [24]. The primary antibodies used were a Purified Mouse Anti-Rat CD43 (W3/13) (BD Biosciences) and anti-CD68 (ED1) (BD Biosciences) antibodies. Histofine simple stain rat MAX-PO (M) was used as the secondary antibody. Twenty glomeruli were selected in random images obtained at low magnification; then, the proportions of glomeruli that exhibited positive staining results for each antibody were determined. Subsequently, results for the *S. mutans* group were expressed as relative ratios; the average values of the control group at each time point were regarded as 1.0. Images were analysed with ImageJ (National Institutes of Health, Bethesda, MD, USA) to determine the ratios of CD43- and CD68-positive areas to whole glomeruli.

### Transmission electron microscopy

For pre-fixation, excised kidney tissue specimens were immersed in a solution of 2% glutaraldehyde and 2% paraformaldehyde in PBS (0.1 M, pH 7.4) for 16–18 h. Post-fixation was then performed in 2% osmium tetroxide for 1.5 h. After specimens had been washed with PBS, they were dehydrated in a graded ethanol series and embedded in low-viscosity resin (Spurr resin; Polysciences, Warrington, PA, USA). Eighty nanometre ultrathin sections were then prepared using an ultramicrotome (EM-UC 7; Leica, Tokyo, Japan); sections were stained with uranyl acetate and lead citrate. Specimens were observed under a transmission electron microscope (H-7650; HITACHI, Tokyo, Japan).

### Statistical analyses

Statistical analyses were performed using GraphPad Prism 8 Statistics Software (GraphPad, Inc., La Jolla, CA, USA). All results are presented as the mean ± standard error. Differences in whole-body weight serum levels, mesangial proliferation scores, and the positive area ratios of CD43 and CD68 were assessed using Student’s *t* test; differences in urinary protein were assessed by Thompson’s rejection test, followed by Fisher’s exact test. In addition, positive immunohistochemical staining results were compared using Fisher’s exact test. *P* values of less than 0.05 were considered statistically significant.

## Results

### Clinical characteristics of rats

**Fig. 1:**
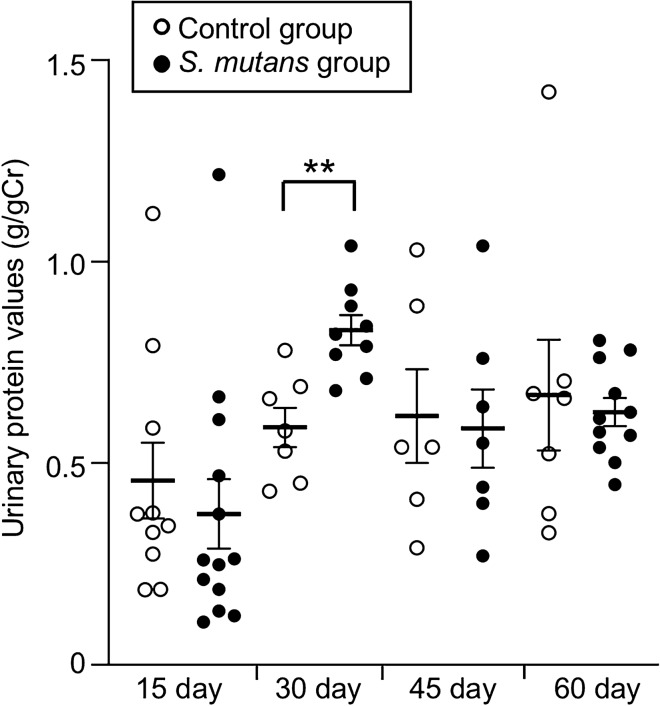
Urinary protein values at each time point. Each column represents the mean ± standard error of 8–12 animals. Statistical significance was determined using Student’s *t* test after Thompson’s rejection test. **P < 0.01

First, we compared the systemic conditions of specific pathogen-free Sprague–Dawley rats that had been administered Cnm-positive *S. mutans* (1 × 10^8^ colony-forming units) isolated from a patient with IgAN (strain SN74; *S. mutans* group) and those of control rats that had been administered PBS (control group). There were no significant differences in whole-body weights between *S. mutans* and control groups throughout the study (Table 1). However, the values of urinary protein at 30 days, standardised to the values of creatinine, were significantly greater in the *S. mutans* group than in the control group (*P* < 0.01) (Fig. 1). In contrast, there were no significant differences between the two groups at 15, 45, and 60 days. Analysis of haematuria (erythrocytes in urine) revealed no significant differences between groups. Table 1 shows the values of representative blood biochemical parameters related to kidney conditions. There were no significant differences in serum albumin level between the two groups throughout the study. However, serum blood urea nitrogen values were significantly greater in the *S. mutans* group than in the control group at 30 days (*P* < 0.05); conversely, there were no significant differences between the two groups in terms of serum blood urea nitrogen values at 15, 45, and 60 days. At 45 days, significantly greater serum creatinine values were observed in the control group than in the *S. mutans* group, whereas there were no significant differences between the groups at 15, 30, and 60 days.

**Table 1:** Whole body weights and serum levels of kidney markers

Days	Whole body weights serum levels	Control group (*N* = 8–10)	*S. mutans* group (*N* = 10–12)	*P *value^a^
15	Whole body weight (g)	209.3 ± 1.8	235.7 ± 9.4	ns
	ALB (g/dL)	3.71 ± 0.08	3.79 ± 0.09	ns
	BUN (mg/dL)	20.38 ± 0.67	18.91 ± 1.12	ns
	CRE (mg/dL)	0.23 ± 0.01	0.24 ± 0.01	ns
30	Whole body weight (g)	410.4 ± 8.1	398.3 ± 5.5	ns
	ALB (g/dL)	3.91 ± 0.02	4.02 ± 0.05	ns
	BUN (mg/dL)	16.50 ± 0.69	18.80 ± 0.58	0.015
	CRE (mg/dL)	0.26 ± 0.01	0.27 ± 0.01	ns
45	Whole body weight (g)	523.4 ± 16.6	521.9 ± 26.2	ns
	ALB (g/dL)	3.70 ± 0.18	3.60 ± 0.17	ns
	BUN (mg/dL)	22.90 ± 1.15	22.20 ± 1.65	ns
	CRE (mg/dL)	0.37 ± 0.02	0.31 ± 0.02	0.048
60	Whole body weight (g)	562.0 ± 8.2	513.9 ± 13.1	ns
	ALB (g/dL)	3.82 ± 0.07	3.87 ± 0.04	ns
	BUN (mg/dL)	21.40 ± 0.75	22.30 ± 0.76	ns
	CRE (mg/dL)	0.37 ± 0.01	0.39 ± 0.02	ns

*P* values of less than 0.05 were considered to indicate significant differences.

^a^Statistical analyses of control and *S. mutans* groups were performed by the Student’s *t* test after Thompson’s rejection test.

### Histopathological and immunochemical findings

**Fig. 2:**
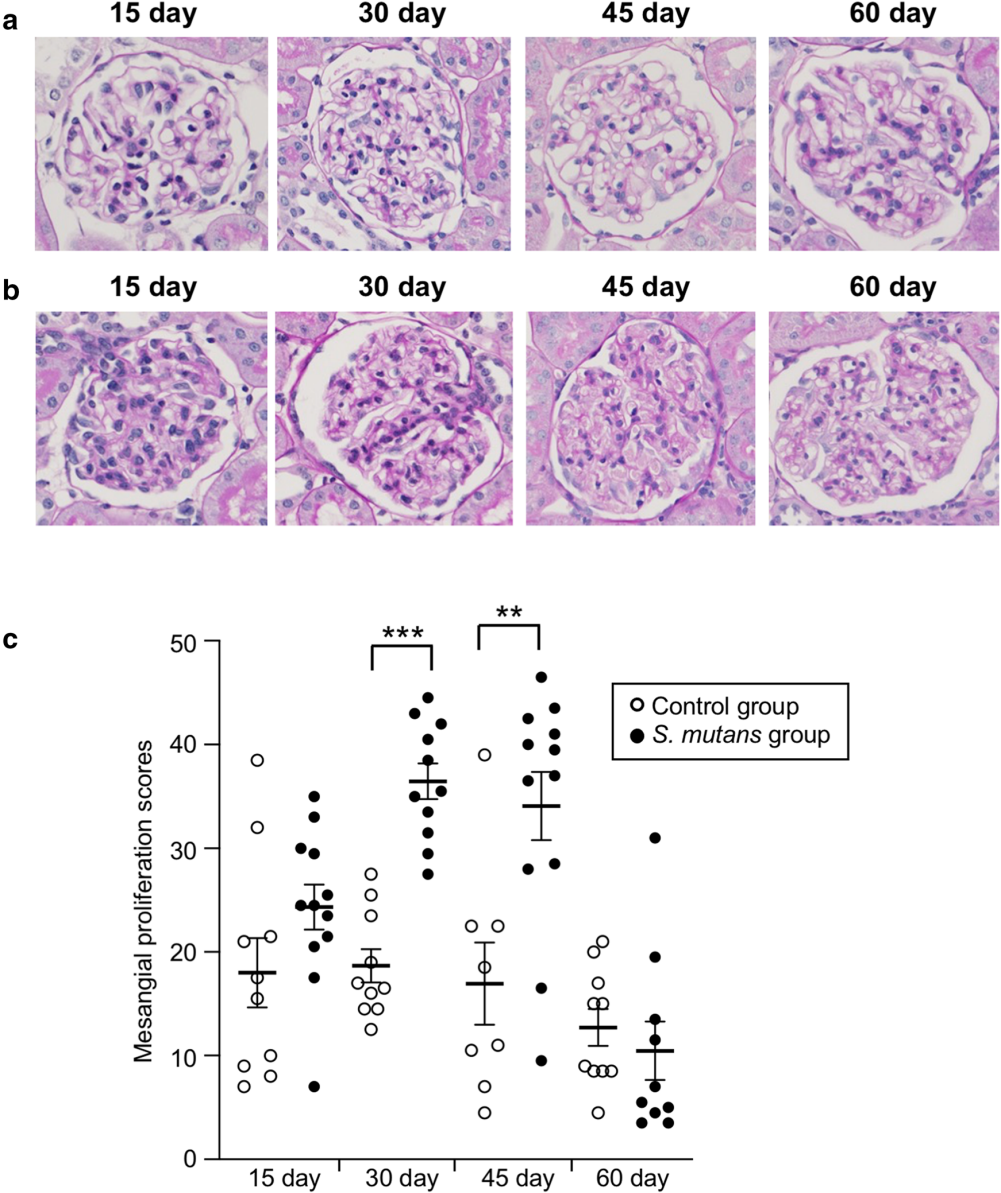
Histopathological appearance of kidney tissues after Periodic acid–Schiff staining. **a**, **b** Representative images of the control group (**a**) and *S. mutans* group (**b**) are shown. **c** Comparison of mesangial proliferation scores between the two groups. Statistical significance was determined using Student’s *t* test. ** *P* < 0.01, *** *P* < 0.001

**Fig. 3:**
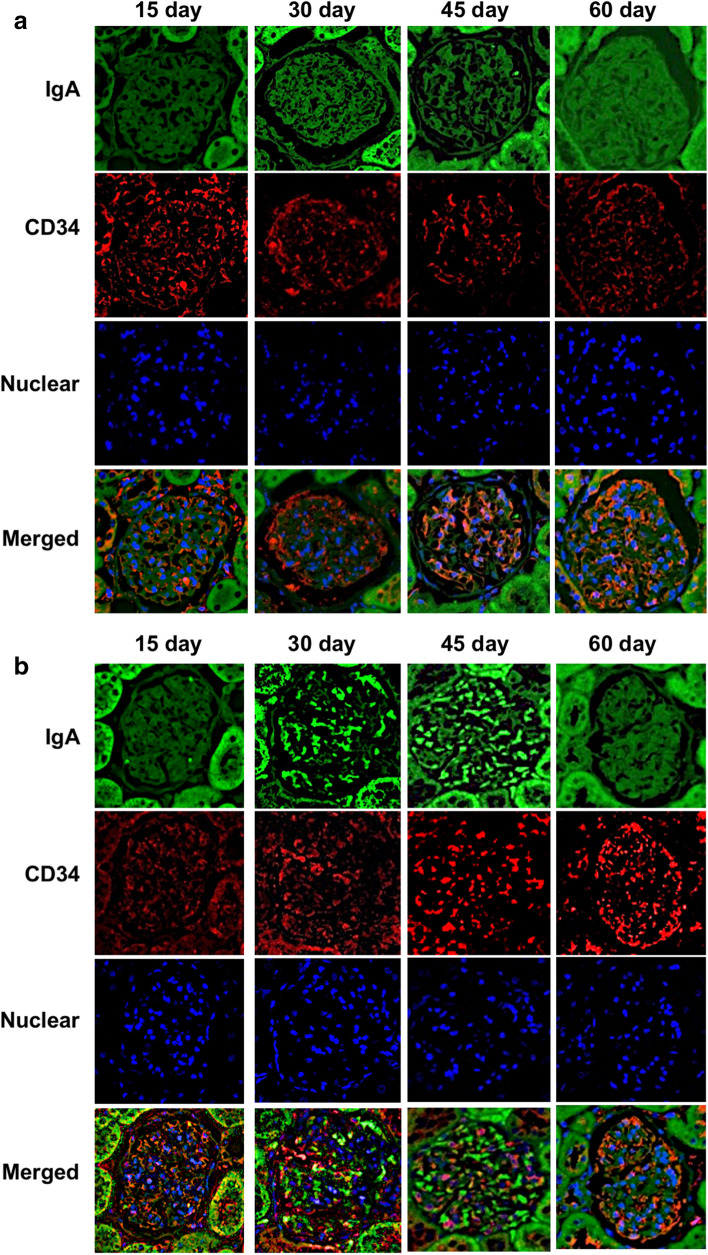
Histopathological appearance of kidney tissues after immunohistochemical staining with an IgA-specific antibody. **a**, **b** Representative images of the control group (**a**) and *S. mutans* group (**b**) are shown

**Fig. 4:**
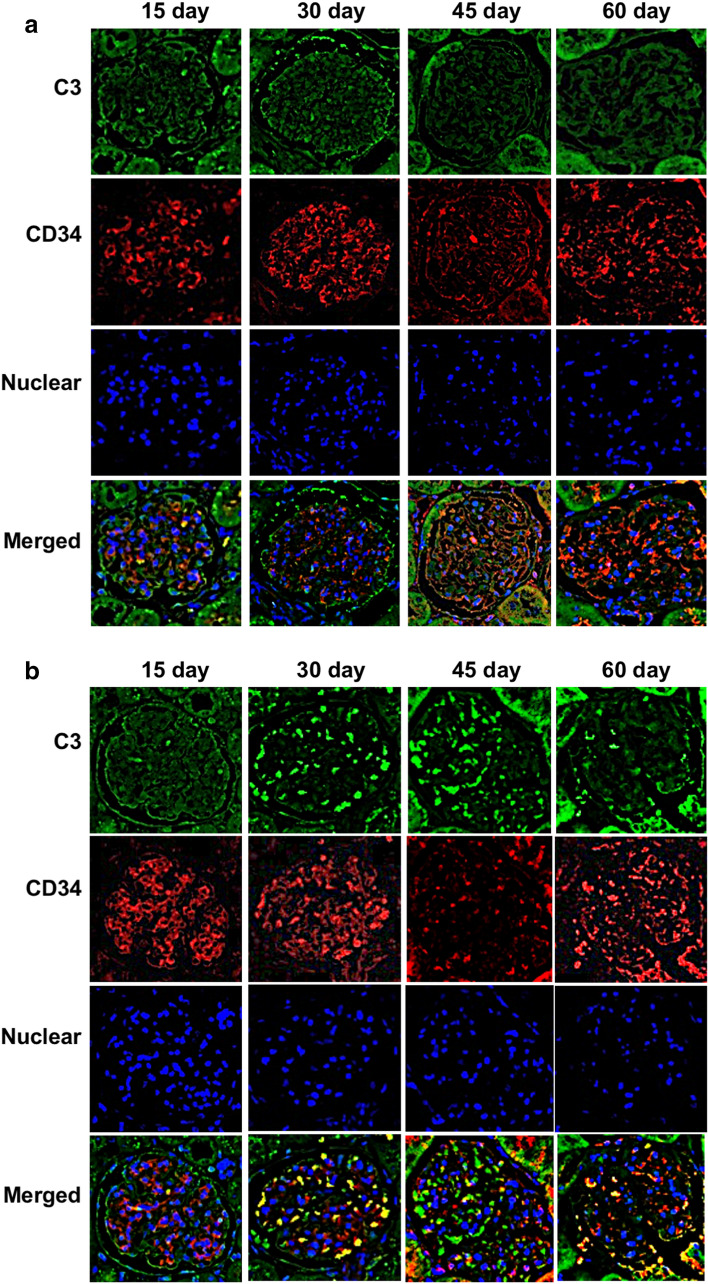
Histopathological appearance of kidney tissues after immunohistochemical staining with a C3-specific antibody. **a**, **b** Representative images of the control group (**a**) and *S. mutans* group (**b**) are shown

Rats were sacrificed at each time point and their kidneys were removed for histopathological examinations. Periodic acid–Schiff staining of kidney sections demonstrated increases in mesangial cell numbers and an increased mesangial matrix at 30 and 45 days in the S*. mutans* group; however, these phenomena were not observed at 15 and 60 days (Fig. 2). In addition, mesangial proliferation scores were significantly greater in the *S. mutans* group than in the control group at 30 and 45 days (*P* < 0.001 and *P* < 0.01, respectively). Notably, there were no significant differences in mesangial proliferation scores between the two groups at 15 and 60 days. Fluorescence immunostaining using an anti-IgA antibody revealed deposition of IgA in the mesangial area of the *S. mutans* group at 30 and 45 days, whereas deposition was not observed at 15 and 60 days (Fig. 3). Fluorescence immunostaining results for CD34 and C3 exhibited partial overlap. Fluorescence immunostaining using an anti-C3 antibody indicated both mesangial and subendothelial deposition of C3 in the *S. mutans* group at 30 and 45 days (Fig. 4). Overall, there were significantly greater proportions of sections with IgA and/or C3 deposition in the *S. mutans* group than in the control group at 45 days (*P* < 0.05); no significant differences were observed at 15, 30, and 60 days (Table 2).

**Table.2:** Positive reactions in immunohistochemical staining

Days	Target(s)	Control group (*N* = 8–10)	*S. mutans* group (*N* = 10–12)	*P* value^a^
15	IgA	0/10 (0%)	0/12 (0%)	ns
	C3	0/10 (0%)	0/12 (0%)	ns
	IgA and C3	0/10 (0%)	0/12 (0%)	ns
30	IgA	0/10 (0%)	4/11 (36.4%)	ns
	C3	0/10 (0%)	3/11 (27.3%)	ns
	IgA and C3	0/10 (0%)	2/11 (18.2%)	ns
45	IgA	0/8 (0%)	7/12 (58.3%)	0.0147
	C3	0/8 (0%)	9/12 (75.0%)	0.0014
	IgA and C3	0/8 (0%)	7/12 (58.3%)	0.0147
60	IgA	0/10 (0%)	0/10 (0%)	ns
	C3	0/10 (0%)	0/10 (0%)	ns
	IgA and C3	0/10 (0%)	0/10 (0%)	ns

*P* values of less than 0.05 were considered to indicate significant differences. ^a^Statistical analyses of control and *S. mutans* groups were performed by Fisher’s exact probability test.

Taken together, the findings indicate that rats administered Cnm-positive *S. mutans* via the jugular vein demonstrated pathological changes suggestive of IgAN-like nephritis at 30 and 45 days, which recovered at 60 days.

### Electron microscopy findings

**Fig. 5:**
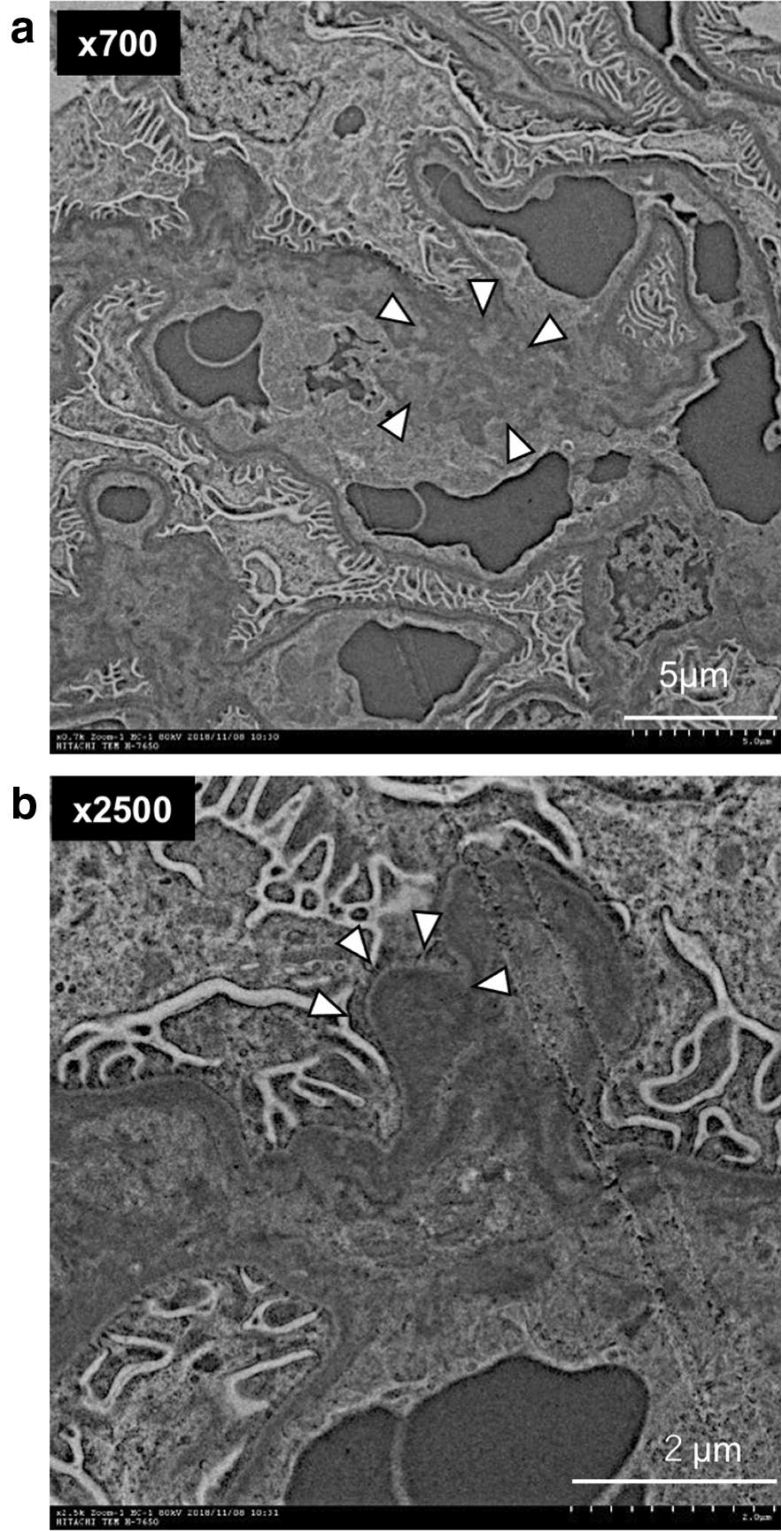
Transmission electron microscopy analyses of kidney tissues. **a**, **b** Representative images of the *S. mutans* group. White arrows indicate electron-dense deposition (**a**) and a hump (**b**)

Electron microscopy analyses demonstrated mesangial deposition in renal glomeruli at 45 days in the *S. mutans* group (Fig. 5). In addition, a hump was observed in the subepithelial region.

### Distribution of immunocompetent cells in the kidney

**Fig. 6:**
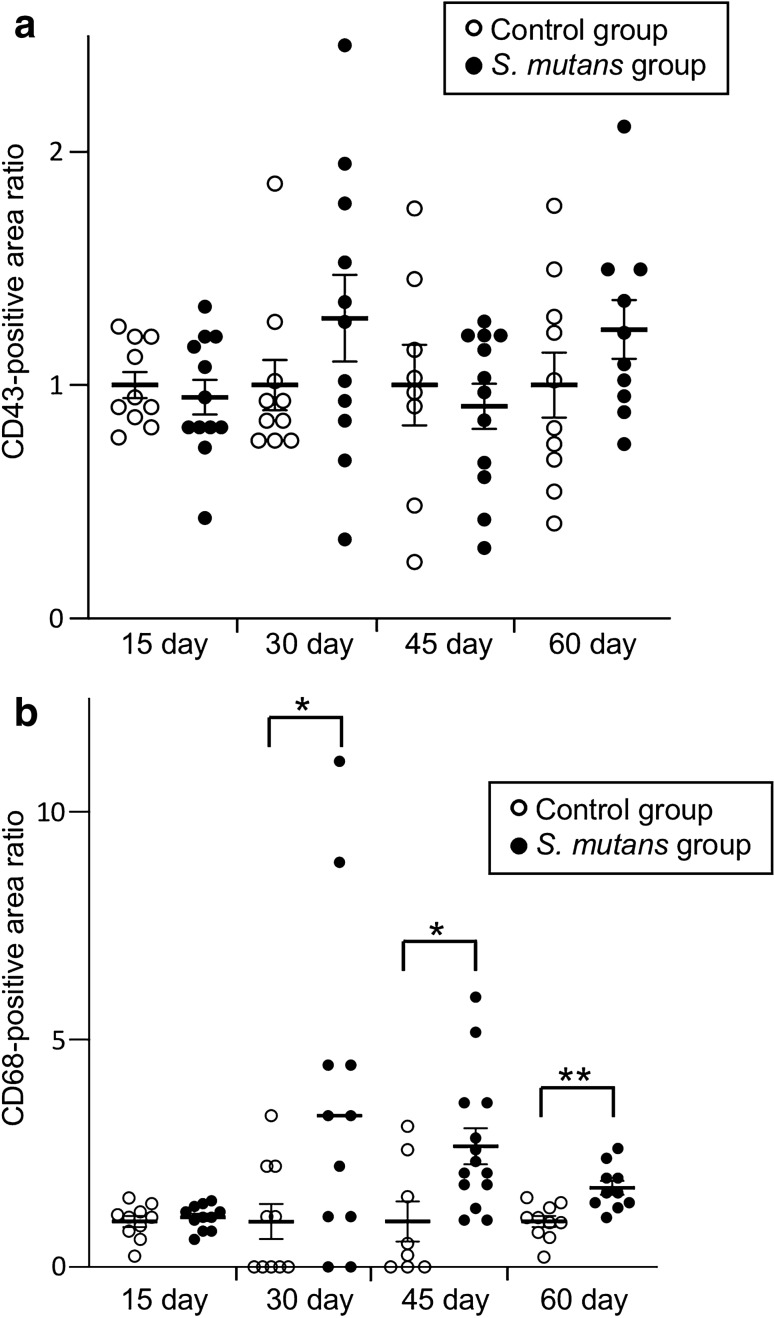
Histopathological appearance of kidney tissues after immunohistochemical staining with a CD43-specific antibody and a CD68-specific antibody. Comparisons of CD43 (**a**) and CD68 (**b**) positive areas between the two groups are shown. Statistical significance was determined using Student’s *t* test. *P < 0.05, **P < 0.01

Immunochemical staining using an anti-CD43 antibody revealed deposition of neutrophils and lymphocytes in glomeruli. There were no significant differences in this deposition between *S. mutans* and control groups during any period (Fig. 6a). In contrast, immunochemical staining using an anti-CD68 antibody demonstrated prominent deposition of macrophages in glomeruli in the *S. mutans* group. The CD68-positive cell proportion was significantly greater in the *S. mutans* group than in the control group at 30, 45, and 60 days (*P* < 0.05, *P* < 0.05, and *P* < 0.01, respectively) (Fig. 6b).

## Discussion

To the best of our knowledge, this is the first study to demonstrate that intravenous administration of *S. mutans* caused transient induction of IgAN-like lesions in rats. The association of *S. mutans* with nephritis was first reported in 1985; intravenous administration of *S. mutans* was found to induce the production of immune complexes, leading to nephritis-like lesions in rabbits [20]. Moreover, a study in 1995 revealed that intraperitoneal administration of *S. mutans* led to elevated serum rheumatoid factor levels, which caused nephritis in rabbits [21]. In terms of animal models of spontaneous IgAN, the HIGA mouse [25] and gddy mouse [26] are well known. However, there have been no reports of IgAN-like lesions in healthy animals that received intravenous administration of bacteria. In the present study, we selected a Cnm-positive *S. mutans* strain for administration to healthy rats, because this type of strain is frequently present in the oral cavity of patients with IgAN [17, 18].

The dose of Cnm-positive *S. mutans* used in this study was determined based on our previous mouse models of cerebral haemorrhage [13], ulcerative colitis [11], and non-alcoholic steatohepatitis [23], as well as a rat model of infective endocarditis [22]. *S. mutans* has been proposed to enter the bloodstream via invasive dental treatment, such as tooth extraction [27]. As expected, IgAN-like lesions were found at 30 and 45 days, but disappeared at 60 days. It is reasonable to speculate that administration of a single intravenous dose of *S. mutans* to healthy rats caused transient induction of the IgAN-like lesions. We found increases in transient proteinuria, mesangial cells, and mesangial matrix in histopathological examinations, mesangial IgA and C3 deposition and partial C3 subendothelial deposition in fluorescence immunostaining, and mesangial deposition and a hump in electron microscopy analyses. C3 deposition was more frequently identified than IgA deposition in rats at 45 days. These features had characteristics similar to those of IgA-dominant infection related to glomerulonephritis (IRGN), in which bacterial infection induces lesions with IgAN-like pathology [28].

It has been reported that neutrophils and macrophages are predominantly found in IRGN, compared with early IgAN; moreover, a reduction of macrophages occurs in IRGN, whereas macrophage infiltration in IgAN continues until the late phase [24]. In the present study, there were no significant differences in CD43-positive cell proportions between *S. mutans* and control groups during any period. In contrast, the CD68-positive cell proportion was significantly greater in the *S. mutans* group at 30, 45, and 60 days; the proportion gradually reduced in the late phase. These results suggest that the lesions in this model have some features similar to those of IgAN in human patients.

A proposed hypothesis regarding the mechanism of onset and development of IgAN involves respiratory sensitisation by bacterial antigens derived from *Escherichia coli, Pseudomonas aeruginosa*, *Haemophilus parainfluenzae*, and methicillin-resistant *Staphylococcus aureus* in the upper airway mucosal epithelium, based on various clinical symptoms in patients with IgAN [6, 29–31]. When bacteria invade the body, the immune defense mechanism is primarily influenced by the bacterial species and bacterial components of the cell wall (e.g., proteins and polysaccharides), which could be important target antigens for antibody reactions [29–31]; Cnm may constitute one of these antigens. The mucosal immune disorder caused by bacterial infection is presumed to induce dysfunction in remote glomerular tissues [6, 32, 33]. Indeed, upper respiratory infection is reportedly frequent in patients with acute IgAN [34]. Thus, persistent contact with a specific type of *S. mutans* could lead to persistent infection that involves overproduction of IgA and production of IgA with abnormal glycans, thereby causing onset of IgAN [35].

In summary, our study demonstrated that the administration of Cnm-positive *S. mutans* caused transient induction of IgAN-like lesions in rats. However, the mechanism underlying these lesions remains unknown and should be investigated in future studies. In addition, bacterial invasion of the bloodstream in the present model may reflect entry during invasive dental procedures (e.g., tooth extraction). Other models of chronic infection by *S. mutans* in rats (e.g., severe dental caries that extend to the pulp space and cause persistent bacteraemia) should be examined in future studies.
